# Implementation evaluation of staff support and wellbeing programmes at an academic health science centre during COVID-19: study protocol

**DOI:** 10.1186/s43058-021-00128-7

**Published:** 2021-02-24

**Authors:** Barbora Krausova, Sam Nishanth Gnanapragasam, Len Demetriou, Alison Beck, Renata Pires-Yfantouda, Mary Jane Docherty, Nick Sevdalis

**Affiliations:** 1 Centre for Implementation Science, Institute of Psychiatry, Psychology and Neuroscience, King’s College London, 16 De Crespigny Park, London, SE5 8AF UK; 2 South London and Maudsley NHS Foundation Trust, Denmark Hill, London, SE5 8AZ UK; 3 Guy’s and St Thomas’s NHS Foundation Trust, Westminster Bridge Road, London, SE1 7EH UK

**Keywords:** Formative evaluation, COVID-19, Coronavirus, Healthcare workers, Staff support, Staff wellbeing, CFIR, EPIS, ERIC

## Abstract

**Background:**

Evidence from previous pandemics as well as early evidence from COVID-19 suggests risk of adverse mental health and wellbeing outcomes for healthcare workers. In response to these concerns, healthcare systems and organisations rapidly established staff support and wellbeing programmes. While there is emerging literature related to the effectiveness of such interventions, what is less well understood and evaluated is the evidence base regarding how such programmes are implemented; what supports and hinders their implementation; and how or if they are maintained following the initial acute phase of the pandemic. This study addresses this gap by studying the implementation process of COVID-19-related staff wellbeing programmes in the three UK NHS Trusts that make up one of Europe’s largest academic health sciences centres, King’s Health Partners.

**Methods:**

We will conduct a prospective, cross-sectional descriptive study using qualitative research methods and non-probability purposive sampling to identify a study participant group representative of the population and implementation activity of interest. We will conduct semi-structured interviews of between 30 min and 1 h. We will identify theory-driven elements in the dataset using the Consolidated Framework for Implementation Research (barriers and drivers), Exploration, Preparation, Implementation, Sustainment Framework (timeline/chronology/evolution of the implementation and different issues at different times) and Expert Recommendations for Implementing Change (implementation strategies). We will then identify indicators of these constructs within the dataset and report them, as well as their inter-relationships.

**Discussion:**

Through this study, we hope to better understand what factors hindered and enabled the implementation of three inter-linked staff support and wellbeing programmes and how/to what extent have these programmes been sustained. We will also explore whether implementation science frameworks are applicable and beneficial in conceptualising and understanding crisis driven and rapidly implemented interventions and in what ways, if any, they need to be adjusted when used in unprecedented circumstances such as the COVID-19 pandemic.

Contributions to the literature

- While there is emerging literature related to effectiveness of staff wellbeing interventions within pandemic contexts, the implementation process that supports early set up and later maintenance of such programmes is not understood.
- This study will offer descriptive evidence on the nature of the barriers and drivers implementors of such programmes face and what implementation strategies were used in the context of a large academic health sciences centre in the UK.
- The study will also explore the utility of existing implementation science frameworks in understanding the very rapid implementation processes due to the COVID-19 pandemic.

## Background

The World Health Organization pandemic preparedness plans have long highlighted the need to provide psychosocial support for healthcare workers (HCWs) as part of the pandemic response [1]. Despite these recommendations existing at the start of the first wave of the United Kingdom (UK) COVID-19 outbreak in March 2020, there was limited planning, evidence and implementation guidance on what this support should look like and who should deliver it.

Pandemics are known to have a significant impact on HCWs. These stem from distinct stressors including exposure to severely ill patients, fear of contagion, longer work hours, physical fatigue, redeployment practices and associated changes in role, disruption of normal supportive structures, separation from families, loneliness and staff retention issues [2–4]. All of these factors can contribute to increased workload and acute stress related to not being unable to cope effectively with external demands. Without intervention, this acute stress response can become chronic and is associated with a range of physiological and psychological impacts such as burnout, occupational stress, and depression [5].

Literature from previous pandemics report a high incidence of acute and post-traumatic stress [6] and longer-term sequelae with potential impact on service provision such as reduced patient contact hours, symptoms of burnout, and behavioural consequences of stress [7, 8]. Early evidence from HCW studies during COVID-19 document a range of adverse psychosocial outcomes including symptoms of depression, anxiety, insomnia and general psychological distress [9–11]. Concerns have also been raised about the risk of moral injury [12]. Reassuringly, there is some evidence that clear communication, access to adequate personal protective equipment, sufficient rest and both practical and psychological support have been associated with reduced HCW morbidity and relatedly that effective interventions are available to help mitigate the psychological distress experienced by healthcare staff in an infectious disease outbreak [6].

It has been reported that healthcare organisations and systems world-wide have undertaken steps to ameliorate the acute stress response and support the mental health and wellbeing of HCWs [4, 13–16]. The nature and extent of these interventions will be variable and likely influenced by local health system resource and pre-pandemic values and attitudes towards staff well-being and mental health. Such programmes have likely incorporated approaches such as increasing access to psychological first aid, adequate rest, peer and social support, building team and organisational resilience [15, 17] and avoiding harmful interventions such as psychological debriefing [18]. More effectiveness studies and evaluations of these COVID-19 responses will certainly be published in the coming months and years.

What is less well understood or evaluated is the evidence base regarding how precisely such programmes were implemented; what supported and hindered their implementation; and how or if they are maintained following the initial acute COVID-19 crisis phase into the medium and subsequently longer term—i.e. whether and how they are sustainable. Such gaps in understanding how complex interventions are implemented tend to exist throughout healthcare and contribute to the so-called ‘implementation gap’. The ‘implementation gap’ refers to the challenge whereby numerous evidenced interventions fail to be successfully implemented routinely and at scale, simply because no one really knows how to do so [19, 20].

### COVID-19-related staff wellbeing interventions within King’s Health Partners (London, UK)

In this study, we will evaluate the implementation of three connected but distinct staff support and wellbeing programmes (SSWP) launched during the COVID-19 pandemic response in King’s Health Partners (KHP) in London (UK), starting in March 2020 and currently still being offered. KHP is a large academic health science centre that includes three National Health Service (NHS) Foundation Trusts: Guy’s and St Thomas’ NHS Foundation Trust (GSTT), King’s College Hospital Foundation Trust (KCH) and South London and Maudsley NHS Foundation Trust (SLaM). KHP provide a range of clinical services—GSTT and KCH are Acute NHS Trusts and SLaM is a Mental Health NHS Trust. A total of 37,870 clinical and non-clinical staff work across these three institutions, providing care for an estimated patient population of eight million.

Each of the three staff support programmes derived from one set of recommendations created from a rapid review of literature and expert consultation during the week of 10 March 2020 at the start of the first wave of COVID-19. The programmes involved multidisciplinary collaborations within each organisation between departments of psychiatry, psychology, occupational health and other stakeholders. Key objectives of the programmes were to meet the acute psychosocial health and wellbeing needs of individual staff members, support staff at work, minimise the impact of the pandemic on staff wellbeing where possible and build structures, cultural change and resource that would remain available for post-outbreak staff needs.

Given the rate of increasing clinical acuity with the evolving first wave of COVID-19 and the impact on staff, the programmes were designed and implemented at significant speed. Approximate roll out time from the first request for staff support to implementation of each core aspect of the programmes occurred in under 4 weeks. Implementation of the first staff support intervention occurred as quickly as 2 days after organisation sign off in one of the organisations. Iterative adjustments to the programmes were made throughout the first wave which lasted approximately 4 months. Work to transition these crisis response initiatives to a next stage response aligning with the organisations’ rest and recovery programmes commenced in June 2020. This transitional work was conducted concurrently with a reduction in staffing of all the different elements of the programmes.

**Fig. 1:**
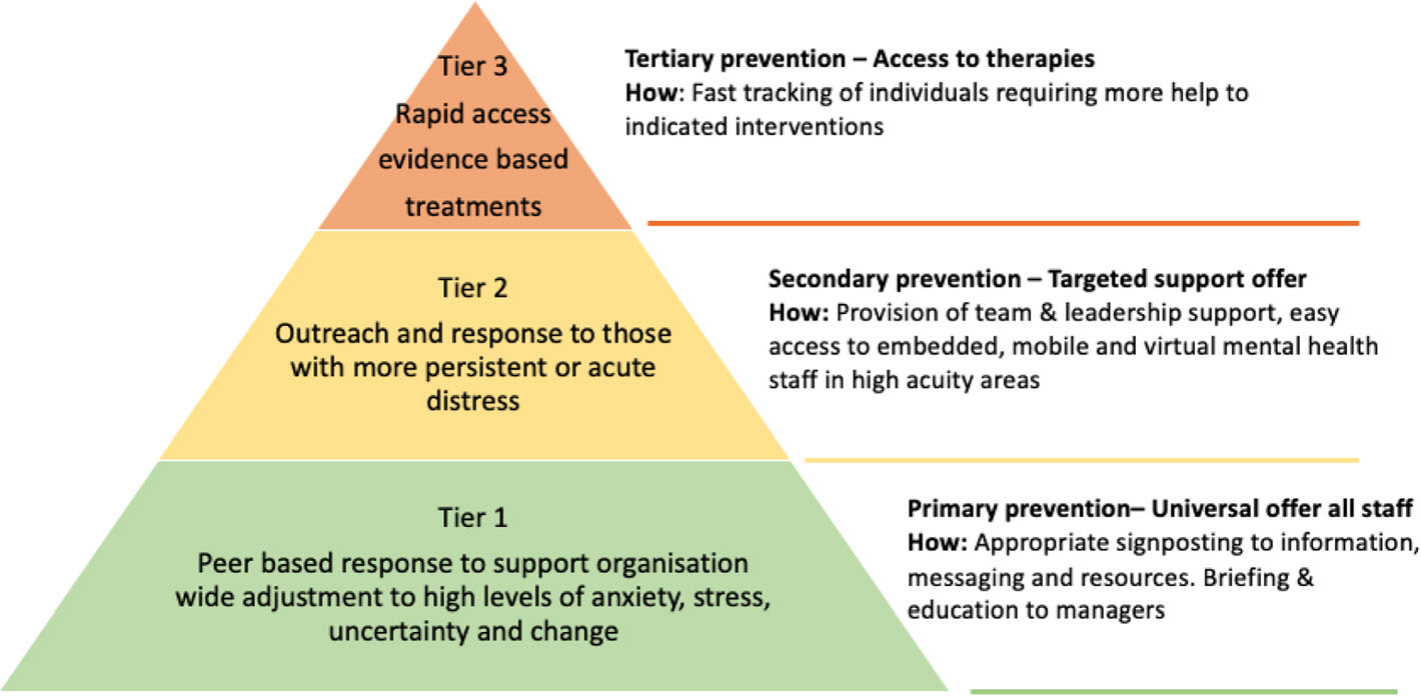
Schematic representation of the tiered prevention strategy applied through the staff support and wellbeing programme (SSWP)

The programme designs involved a tiered and targeted framework for staff support, created to provide primary, secondary and tertiary levels of intervention depending on the level of need (Fig. 1). This tiered model recognised that access to psychological interventions would need to co-exist alongside an organisational-wide effort to build resilience and mental health literacy in psychologically healthy people (Tier 1). Several interventions were developed in each organisation to deliver the tiered model. The exact interventions delivered differed between organisations determined by pre-existing structures and resource. These included:

- Resources and interventions to increase self-care, self-management and mental health literacy and to meet practical needs (food, rest, social connection and peer support).
- Resources and interventions to support leadership and team functioning including uptake and implementation of staff support and well-being practices within teams and departments.
- Rapid access to psychological support including onward referral to more specialised or formal mental health care as required, e.g. through embedded mental health experts in the hospitals or based remotely and accessible through phone or videoconference off site.

Methods to access these tiers of support and to build resilience were then targeted strategically towards key operating units of the organisation namely individuals, teams and leaders using a range of different strategies.

### Study aims

The study will address the following research questions:

1. What factors hindered (barriers) and enabled (facilitators) the implementation of three inter-linked staff support and wellbeing programmes in three London NHS Trusts during the first wave of the COVID-19 pandemic? Sub-question: how and to what extent have these programmes been sustained since?
2. Are implementation science frameworks applicable and useful in conceptualising and understanding crisis-driven and rapidly implemented interventions, such as the staff support and wellbeing programmes in this study? Sub-question: in what ways, if any, do they need to be adjusted when used in unprecedented circumstances such as the COVID-19 pandemic?

The research questions will be answered by addressing the following objectives:

i. Identification of the implementation strategies used to introduce the staff wellbeing programmes across the three hospitals. This will allow determination of which strategies might have been more successful to-date and identify which components of the programme were delivered, and how, across the different Trusts.
ii. Analysis of whether/how these strategies have changed as the implementation progressed from initial crisis response into transitional and more medium-term forms.
iii. Appraisal of what has remained/been sustained of the staff support programmes since inception to-date and the perceptions of staff regarding the need for such programmes to exist (and in what format) in the future.
iv. Identification of lessons and recommendations for informing policy makers, managers and providers on developing, implementing and sustaining staff support and wellbeing programmes during a pandemic or other major incident.
v. Application of three implementation science frameworks (see Methods) to data collection and analysis throughout the study to inform, structure and interpret the study findings.

### Methods and study design

This will be a prospective, cross-sectional descriptive study using qualitative research methods. Qualitative methods will be utilised in order to elicit detailed views, narratives and experiences of the staff involved in designing and implementing the SSWP. Conceptually, the study is a formative evaluation [21], designed in a partnership between the designers and organisational leaders of the staff wellbeing programmes across KHP Trusts (MD, RPY, AB), a team of implementation/improvement scientists (BK, NS) and clinicians (SG). The study is designed in a manner that will inform the subsequent development and maintenance of the staff wellbeing from 2021 onwards. Theoretically, the study will be informed by a number of well-established implementation frameworks, which will be used to frame the data collection and analysis and offer a theory-informed lens on the implementation process. The following frameworks will be applied:

The Consolidated Framework for Implementation Research (CFIR), https://cfirguide.org/ [22], allows mapping of barriers an intervention may face in its implementation across five different but interlinked elements. These include the characteristics of the intervention itself (e.g. how well evidenced, how complex), the individuals involved in the implementation process (including funders and implementors), the process of implementation (including organisational support structure, the presence of an implementation strategy or manual), the inner organisational setting (i.e. the organisational priorities, support and climate across the NHS Trusts implementing the staff support programmes in the context of this study) and also the outer setting (i.e. wider external NHS and pandemic context in this study). CFIR is ideally suited to the exploration of barriers and drivers/facilitators to an implementation process. We will apply the framework to help identify questions to ask through interviews and to facilitate the identification of stakeholders.

We will also apply the Exploration, Preparation, Implementation, Sustainment (EPIS), https://episframework.com/ [23], framework alongside CFIR. The two frameworks are compatible, in that they share the elements of inner and outer context of the intervention implementation. The additional value of applying EPIS is that it offers a time lens to the implementation process: it considers factors affecting the implementation from initial inception of the programme (Exploration phase), to the Preparation phase for the early implementation—often undertaken as a pilot phase, with an element of trial-and-error involved. This is subsequently followed by the Implementation phase, where the programme is fully delivered, and finally, the framework allows consideration of the Sustainment phase, which effectively reflect medium to longer-term programme sustainability. We will use EPIS to determine the historical evolution of the wellbeing programme implementation, from its original inception through to the current implementation—and with a view to establish what is required for future sustainability of the programme elements that are deemed to be sustained beyond the pandemic.

Further to these, we will apply a practical taxonomy of existing implementation strategies used within healthcare to identify and comment on the strategies planned and subsequently used for the early, ongoing and future wellbeing programme implementation. These will be mapped onto the ERIC (expert recommendations for implementing change) framework of implementation strategies [24]. The framework includes nine themed categories of strategies that can be used to introduce a novel programme—including strategies focused on stakeholder engagement, use of evaluation and feedback techniques, education and training and others. The taxonomy has been developed for healthcare based on systematic review of evidence and expert consensus, and it has been shown to be predictive of implementation success in the context of infectious diseases management (e.g. the more strategies used the more successful the uptake of Hepatitis-C treatments in published USA studies). The taxonomy will be used to analyse interview data and theme the strategies that will emerge from the study participants’ interviews. We will map these strategies across the CFIR barriers (i.e. we will seek to determine how the selected strategies addressed programme implementation barriers [25]), and we will also assess whether different strategies were used at different points of the programme implementation (i.e. across the EPIS chronology).

### Participants and recruitment

The study population will comprise staff employed by or affiliated to one or more of the KHP Trusts and involved in the design, implementation and/or delivery of staff support and wellbeing programmes during the first wave of the COVID-19 pandemic (i.e. from March 2020). No limitations will be made based on gender, age, ethnicity, socio-economic group, clinical/non-clinical role and occupation.

We will be using non-probability purposive sampling to arrive at a participant group that can be logically considered to be representative of the population and implementation process of interest.

Stakeholder analysis will be carried out to identify key individuals [26]. This analysis will be based on existing institutional knowledge of the study investigators, some of whom were leading the development of the SSWPs, and relevant documents. Documents considered will include institutional management structures and those directly related to the SSWPs.

We will identify and invite staff across different levels of involvement, who will be categorised as ‘Executive’—staff with executive powers authorising the programmes, ‘Leadership’—senior staff leading the design and development of the programmes and ‘Delivery’—staff delivering specific interventions within the programmes. Some participants might hold a dual ‘Leadership and delivery’ role where they were both leading the programme’s development and delivering some of the interventions.

We will ensure different staff groups (clinical and non-clinical) are represented from a range of departments and disciplines including psychologists, counsellors, liaison psychiatrists, programme and project managers, occupational health staff, organisational development staff and volunteer workers.

Key individuals identified (prospective participants) will be contacted via email by study investigators in the first instance. In this contact, a participant information sheet will be provided to explain the study and what participation would involve. It will be made clear that participation is voluntary and that they may withdraw from the study without giving a reason at any point. Written informed consent (e-form) will be obtained for those wishing to participate in the study.

Sample size is guided by current recommendations for qualitative studies, which suggest that saturation in the themes that emerge through successive interviews can be reached with a minimum of six to twelve participants [27]. We therefore estimate that in order to reach saturation given the heterogeneity of the interventions, as well as similarities and differences across the three NHS Trusts, we will need a minimum of 36 participants (12 per Trust). We propose the following distribution: 1–2 participants in the Executive category in each Trust, 4–5 participants in the Leadership category in each Trust, and 5–6 participants in the Delivery category in each Trust. This distribution reflects the inherent pyramid-shaped distribution of roles in the organisations where there are only a small number of staff with executive powers (on top the pyramid) and there are many more staff working on the ground delivering interventions (at the base of the pyramid).

### Data collection

Semi-structured interviews of between 30 min and 1-h duration will be conducted virtually. A topic guide, which will be shared with participants prior to their interview, will be used and this will be tailored to the three categories of participants (Executive, Leadership or Delivery) to reflect their different roles on the programmes. However, the same key themes will be explored with all participants including organisational context, such as participants’ views on the organisation’s preparedness to meet the support needs of its staff, and exploration of organisational structures and processes that might have helped or hindered the staff support implementation efforts. The topic guides are developed and piloted iteratively by all authors, hence including clinical, implementation/leadership and implementation perspectives and can be viewed in Supplementary Information, Additional file 1. All interviews will be conducted using Microsoft Teams video conferencing software.

### Data analysis

Interviews will be recorded for transcription purposes using the in-built recording feature of the Teams software and a backup recording made on a Dictaphone. Recordings will be transcribed using Otter, a speech-to-text application, which will be subsequently checked for accuracy by the research team who will remove all personally identifiable information to protect the participants’ identity. All interview recordings will be destroyed after the transcription process has been completed.

Interview transcripts will be analysed thematically, using a mixture of inductive and deductive techniques. We will identify theory-driven elements in the dataset using CFIR (barriers and drivers), EPIS (timeline/chronology/evolution of the implementation and different issues at different times) and ERIC (implementation strategies). We will identify indicators of these constructs within the dataset and report them, as well as their inter-relationships. We will also explore and highlight themes that might emerge from the dataset that the implementation frameworks do not necessarily cover. These will be fed into the overarching thematic structure that will emerge from the dataset and reported alongside the theory-driven elements. We will develop a thematic structure, informed by the theoretical constructs as they manifest themselves within the dataset and further enriched and refined by any additional themes and relationships emerging from the inductive analyses.

We will tabulate the thematic structure and provide illustrative quotes that demonstrate the constructs and emergent themes. We will organise the data and track themes using Microsoft Excel.

## Discussion

This study seeks to understand the implementation process of designing, developing, delivering, and sustaining complex multi-component SSWPs in three London Trusts during the first wave of the COVID-19 pandemic, in order to add to an important but as yet limited evidence base regarding this topic. In this context, there was a substantial volume of expert opinion and guidance produced in a short space of time on staff support responses, often drawing on experience in past pandemics or major incidents but without accompanying information on how to implement suggested interventions into varied and complex health care settings [28]. These omissions remain a significant limitation of the current evidence and knowledge base in this field. Understanding the implementation experience of recent efforts will be essential to inform future work including policy guidance and service planning advice during ongoing waves of COVID-19, preparation for other major incidents including future pandemics requiring rapid mobilisation of staff support, and in implementing SSWPs outside of crisis contexts.

Understanding how sustainable these responses were and the factors that influenced should be of particular interest to providers, commissioners, and policy makers. It will be important to understand if the recent surge of interest in the health and wellbeing of the health and care workforce that led to the development of SSWPs was sustained outside of the crisis response and what factors influenced this.

This study also provides an opportunity to examine the unique circumstances COVID-19 presented for implementation science. The field of implementation science is rife with theories and frameworks—some based on substantial psychological, sociological, and organisational science theories, others more narrowly focused on the process of implementation and its determinants. Reviews of theories and frameworks in the field have identified several dozen such theories [29, 30]—yet they are often not used in the context of implementation or applied superficially [29]. Whereas some of these are well supported by evidence and used in numerous studies across a range of healthcare settings, the rapid advent of the COVID-19 pandemic poses significant questions regarding the utility and applicability of the constructs and processes implementation theories posit in relation to implementing evidenced interventions. Early theoretical discussions and viewpoints have started to consider the applications and challenges of utilising implementation science during a scenario such as COVID-19 [31, 32] and called for the need to develop new perspectives and approaches in the field [33]. We currently however lack direct evidence regarding how well theories fare in this type of context and how useful they can be in terms of supporting evaluations of aspects of the COVID-19 response. This study will provide an opportunity to start closing this gap by examining their direct application and limitations in this context and increase understanding of the rapid implementation processes and criteria that the pandemic necessitated.

As this study is being undertaken during the ongoing COVID-19 pandemic, this raises a number of practical issues to consider: Due to social distancing guidelines, all data collection will take place virtually. This might on occasion present technical challenges such as unreliable internet connection or software problems. We will limit the impact of these by always ‘double recording’, i.e. using a separate Dictaphone in addition to recording within the video-conferencing software itself. Where possible, we will also have two researchers conducting each interview, so if one researcher experiences technical difficulties, the interview can carry on.

A different challenge posed by virtual interviewing is that of an altered dynamic or rapport between the interviewer and participant. For instance, we will not have some of the usual interactional prompts and signals such as body language or nonverbal cues. A virtually conducted dialogue might also flow less ‘naturally’ due to time lapses in transmission/internet connection. However, over the course of this year, most people will have experienced numerous if not daily virtual interactions, both formal and informal, and this will not be a novel or unexpected situation for those involved. We will endeavour to make participants feel comfortable and at ease, for instance by giving them the choice to switch the camera (video) on or off during interview as they prefer.

Finally, we acknowledge and anticipate that the people we wish to speak to will continue to be busy and likely working under highly stressful circumstances due to the ongoing nature of the pandemic. This might have a bearing on participant availability (to take part) but also the mental energy that this might require. In addition, we are aware that a second or further waves of the pandemic might be occurring at the time of our study and this will be further compounded by the traditionally busy and challenging period for health and care services during winter months. We will remain vigilant and mindful of these issues and adjust recruitment and data collection accordingly. For instance, if a number of participants indicate that they would like to take part but do not have the time during our pre-specified/preferred time frame, we will need to adjust our time frames to suit the needs of the participants.

We recognise that this study has inherent limitations. Firstly, the study is overall of small scale, due to its qualitative nature, and carried out within a single academic health sciences centre. As such, the generalisability of the findings will be limited to centres and hospital of similar context. Secondly, we cannot rule out self-selection bias in participant recruitment (which is entirely voluntary) and social desirability affecting the data they generate. Care is taken such that the interviewers are distanced from the organisational structures and hierarchies; this lowers but does not eliminate the possibility of these biases. Thirdly, the study is assessing what is, in many ways, a moving target: as the COVID-19 pandemic continues to evolve and KHP Trusts continue to respond to it, the staff wellbeing programmes will likely continue to evolve in response to the pandemic challenges and concurrently with the study. This means that some of the study findings (those that relate to programme implementation and future planning) may be superseded by the time the study is fully completed. The study also has strengths—including its theoretical underpinnings (both clinically and also from the perspective of implementation theory), its formative nature, and the strong partnership between implementors and evaluators, which will facilitate uptake and practical use of the study results within the three Trusts and KHP.

## Data Availability

The datasets used and/or analysed during the current study will be available from the corresponding author on reasonable request.

## Abbreviations

SSWPs: Staff support and wellbeing programmes
KHP: King’s Health Partners
UK: United Kingdom
NHS: National Health Service
CFIR: Consolidated Framework for Implementation Research
EPIS: Exploration, Preparation, Implementation, Sustainment framework
ERIC: Expert Recommendations for Implementing Change framework
COVID–19: Coronavirus disease
HCWs: Healthcare workers
GSTT: Guy’s and St Thomas’ NHS Foundation Trust
KCH: King’s College Hospital NHS Foundation Trust
SLaM: South London and Maudsley NHS Foundation Trust
